# MutMap-Based Cloning of a Soybean Mosaic Virus Resistance Gene

**DOI:** 10.3390/plants14223504

**Published:** 2025-11-17

**Authors:** Bin Wang, Xiaofang Zhong, Debin Yu, Demin Rao, Lu Niu, Hongwei Xun, Xiangyu Zhu, Lu Yi, Xueyan Qian, Fangang Meng

**Affiliations:** 1 Jilin Academy Agricultural of Science, Northeast Agricultural Research Center of China, Changchun 130033, China; wangbin@jaas.com.cn (B.W.); xfzhong649@163.com (X.Z.); yudebin@jaas.com.cn (D.Y.); raodemin@jaas.com.cn (D.R.); niulu@jaas.com.cn (L.N.); 2 Key Laboratory of Molecular Epigenetics of MOE, Northeast Normal University, Changchun 130024, China; xunhw334@nenu.edu.cn; 3 Jilin Provincial Agricultural Technology Extension Station, Changchun 130062, China; tgzzhuxy@163.com (X.Z.); tgzyinlu@163.com (L.Y.)

**Keywords:** soybean, SMV, EMS, MutMap, resistance gene, *Glyma.13G194900^M^*

## Abstract

Soybean is rich in protein and oil and serves as the most important legume crop globally. Soybean mosaic virus (SMV) is a severe threat to soybean production worldwide. MutMap, a gene-mapping technology based on map-based cloning and whole-genome resequencing, is utilized to clone key regulatory genes for agronomic traits in plants. However, no relevant studies have reported the cloning of genes resistant to SMV. We used an M3 mutant population derived from ethyl methanesulfonate mutagenesis of Williams 82, and conducted field inoculation experiments involving the SMV-SC3 strain. After field validation, two lines with high resistance to SMV were finally identified. Using MutMap, we initially screened candidate genes for SMV resistance and found that the G-to-A transitions of one candidate resistance gene, *Glyma.13G194900*, were at base positions 122 and 166. These transitions resulted in the substitution of glycine with glutamic acid (GGA→GAA) and valine with aspartic acid (GTT→GAT), respectively. Transgenic functional validation in soybean showed that the mutant allele of *Glyma.13G194900* (designated *Glyma.13G194900^M^*) substantially enhanced resistance to SMV-SC3, in contrast to the wild-type allele, which did not enhance resistance. Our results demonstrate that MutMap can rapidly identify SMV resistance-related genes to provide a genetic resource that accelerates the breeding of new SMV-resistant soybean.

## 1. Introduction

Soybean (*Glycine max* (L.) Merr.) is a primary source of protein and oil for humans and livestock [1]. Soybean mosaic virus (SMV) is one of the most destructive pathogens affecting soybean production across countries [2], and it is transmitted by aphids and seeds [3]. Upon SMV infection, the leaves of susceptible soybean plants exhibit both mosaic and necrotic symptoms. Early in infection, mosaic symptoms first appear as green spots on young leaves, but as the disease progresses, the leaves undergo morphological changes such as shrinkage. By contrast, necrotic symptoms in the early stage are characterized by brown lesions on newly emerged trifoliate leaves or necrosis in areas such as leaf veins. As necrotic areas expand, the affected leaves eventually become fully necrotic and abscise [4]. SMV infection severely impairs soybean yield and seed quality. Under natural conditions, it causes a soybean production yield loss of 8–50% in China. The most economical and effective strategy used to control SMV in production is to select and cultivate disease-resistant cultivars. However, during the interaction between SMV, hosts, and the environment, mutations in and recombination between different SMV physiological races have driven the rapid evolution of new strains, leading to resistance loss of originally resistant soybean varieties [5].

SMV strains are classified based on their pathogenic phenotypes in different soybean cultivars. In the United States, 98 SMV isolates have been classified into seven strains (G1–G7) using eight differential soybean cultivars [6,7], whereas in China, 22 strains (SC1–SC22) have been identified based on their responses to ten soybean cultivars [8,9]. The recognition of a specific viral protein by a host resistance (R) protein mediates disease resistance. Three SMV resistance loci (*Rsv1*, *Rsv3*, and *Rsv4*) have been identified in soybean, located on chromosomes 13, 14, and 2, respectively [10,11,12]. The *Rsv1* locus confers resistance to SMV strains G1–G3 but susceptibility to strains G5–G7 [13]. In contrast, the *Rsv3* locus provides resistance to strains G5–G7 but not to G1–G4 [14]. The *Rsv4* locus confers resistance to all known SMV strains at the seedling stage, although systemic symptoms often develop in mature plants [15]. To date, several SMV resistance-related genes have been cloned, including *GmPAP2.1* [16], *GmPEX14* [17], *GmAKT2* [18], *GmPP2C3a* [19], *GmCnx1* [20], *GmeEF1a* [21], *GmSN1* [22], GmKR3 [23], *GmCYB5* [24], *GmF3H1* [25], *GmF3H2* [25], *GmFNSII-1* [25], *GmST1* [26], *Rsc4-3* [27], *GmCAL* [28], *GmTOC1b* [29], *GmGSTU23*, *GmGSTU24* [30], *RSC3K* [31], *Gm18GRSC3* [32], and *GmCSDs* [33].

Forward genetics is a powerful approach used to identify genetic mutations that underlie phenotypic variation in plants. In conventional forward genetics-based gene cloning, researchers typically construct complex genetic mapping populations and linkage maps. Subsequent linkage analysis using molecular markers helps pinpoint major genes that control mutant traits. As many phenotypic traits are controlled by multiple loci, the resolution of gene mapping depends heavily on the density of available molecular markers for genotyping. Although forward genetics has been widely used for over 50 years, it requires substantial time and labor, which has limited its broader application [34]. The development of advanced DNA sequencing technologies in the 21st century has significantly reduced sequencing costs. In 2012, scientists developed MutMap, a breakthrough method that combines breeding and sequencing strategies: wild-type plants are mutagenized with ethyl methanesulfonate (EMS) to generate mutants, which are then selfed for several generations to achieve homozygosity; homozygous mutants are crossed with the wild type to produce F_1_ hybrids, and F_1_ plants are selfed to obtain an F_2_ population with segregated traits. Individuals in the F_2_ generation with phenotypes distinct from the wild type are selected for bulk sequencing, with the wild-type parent sequenced as a reference. Genomic regions with SNP-index (the proportion of reads containing a SNP (Single-nucleotide polymorphism) relative to the reference) that is significantly higher than the expected background level are identified as candidate causal loci [35]. MutMap has been successfully used to clone key regulatory genes for agronomic traits in many species, including rice and Arabidopsis [36].

Despite its large genome, soybean is amenable to MutMap analysis, as its reference genome sequence was released in 2010 and it is easy to hybridize [37]. However, its application to cloning SMV resistance genes has not been reported. Therefore, in this study, we used MutMap to identify *Glyma.13G194900^M^* as a major candidate SMV resistance gene, which is a gain-of-function resistance gene. Its function was further confirmed via transgenic validation, showing high resistance to the SMV-SC3 strain. This integrated strategy facilitates the identification of key resistance genes for SMV.

## 2. Results

### 2.1. Identification and Evaluation of SCV Resistance in EMS-Induced Mutants

**Figure 1:**
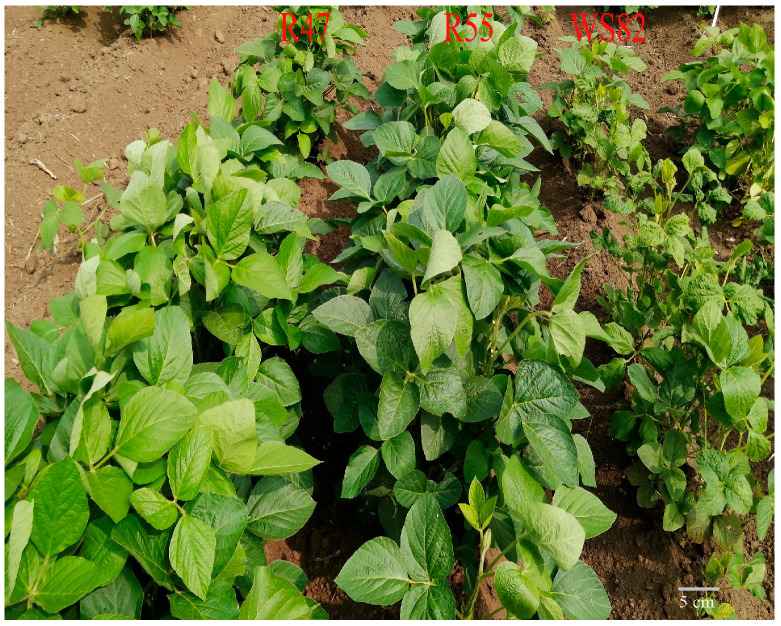
Resistance evaluation of the highly resistant lines R47 and R55, derived from EMS mutagenesis of the susceptible cultivar Williams 82, was conducted via field inoculation with SMV-SC3. For the M_4_ generation of R47 and R55, 15 plants per row were planted with three biological replicates. In the field experiment, these plants were inoculated with SMV-SC3 at 21 and 30 days after emergence, and phenotypes were observed one month post-inoculation.

A total of 14,000 EMS-mutagenized M_3_ seeds of the soybean cultivar Williams 82 were sown in the experimental field in Gongzhuling, Jilin Province (Figure S1). Disease resistance was evaluated via successive inoculations with SMV-SC3 at three growth stages: when the first trifoliate leaf was fully expanded, and at 40 and 50 days after emergence. Phenotypic screening showed that most lines exhibited susceptible symptoms, and susceptible plants exhibited leaf wrinkling, mosaic patterns, stunted growth, and delayed development. In contrast, highly resistant plants grew normally and showed no visible disease symptoms (Figure S1). Ultimately, 12 highly resistant individual plants were identified from the EMS mutant population. These putative resistant plants were re-evaluated for SMV resistance in the following year, and two resistant lines (R47 and R55) with high resistance to SMV-SC3 were selected (Figure 1). The resistant lines consistently exhibited a highly resistant phenotype, whereas wild-type plants showed typical SMV symptoms, including leaf mosaic, shrinkage, and seed mottling.

### 2.2. MutMap-Based Mapping of Resistance Candidate Intervals

To map the resistance loci of the two highly resistant lines (R47 and R55), we crossed the R47 and R55 mutants with wild-type Williams 82, obtaining 8 and 10 F_1_ hybrid seeds, respectively. The F_1_ plants were self-pollinated to generate F_2_ populations, *and these* were then evaluated for resistance to SMV-SC3 under field conditions. A segregation analysis of resistant and susceptible plants in the F_2_ generation (R47:580 resistant: 187 susceptible, χ^2^ test = 0.157, *p* = 0.692; R55:350 resistant: 114 susceptible, χ^2^ test = 0.046, *p* = 0.830) indicated that resistance in both mutants was controlled by a single dominant gene.

**Figure 2:**
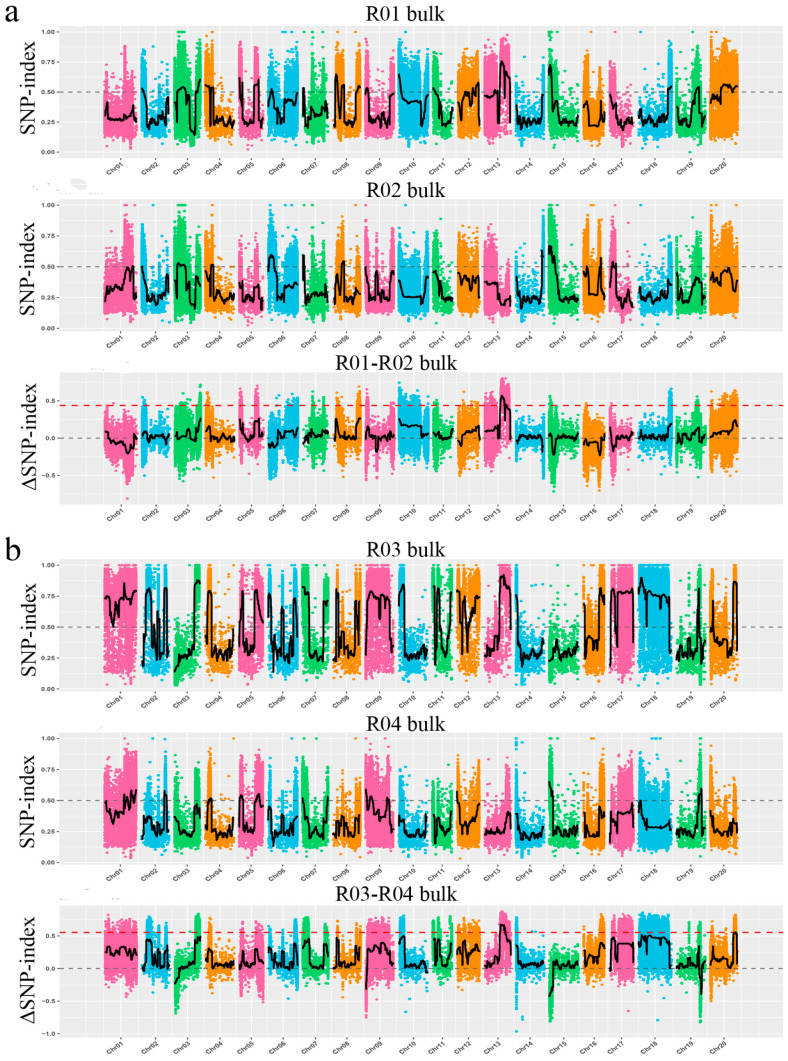
Distribution of SNP-index and ΔSNP-index across 20 chromosomes in the highly resistant lines R47 and R55. The dashed line indicates the significance threshold for the SNP-index, while the solid black line shows the actual SNP-index values across chromosomal positions. The alternating colors represent the distribution of the SNP-index on different chromosomes. (**a**) Distribution of SNP-index and ΔSNP-index across the 20 chromosomes in the resistant line R47. R01 bulk represents a pool of 50 resistant individual plants, and R02 bulk represents a pool of 50 susceptible individual plants. (**b**) Distribution of SNP-index and ΔSNP-index across the 20 chromosomes in the resistant line R55. R03 bulk represents a pool of 50 resistant individual plants, and R04 bulk represents a pool of 50 susceptible individual plants. The X-axis represents the chromosomes, and the Y-axis indicates the SNP-index and ΔSNP-index values, respectively. Genomic regions with a ΔSNP-index greater than 0.5 were designated as candidate regions.

For each F_2_ population, 50 susceptible and 50 resistant plants were selected to form a susceptible pool and a resistant pool for bulk segregant sequencing. Single-nucleotide polymorphism (SNP) calling was performed using GATK. Based on the reference genome, SNPs were identified and filtered, and SNP-index values for each SNP in the parents and pooled samples were calculated. We used the recently published ΔSNP-index method to conduct a marker-trait association analysis based on genotype frequency differences between parents and pooled samples. We calculated the ΔSNP-index for all 20 soybean chromosomes, and regions with a ΔSNP-index > 0.5 were designated as candidate regions (Figure 2). The results showed that the candidate regions for both R47 and R55 are located on chromosome 13, with a significant overlapping segment (Figure 2).

**Figure 3:**
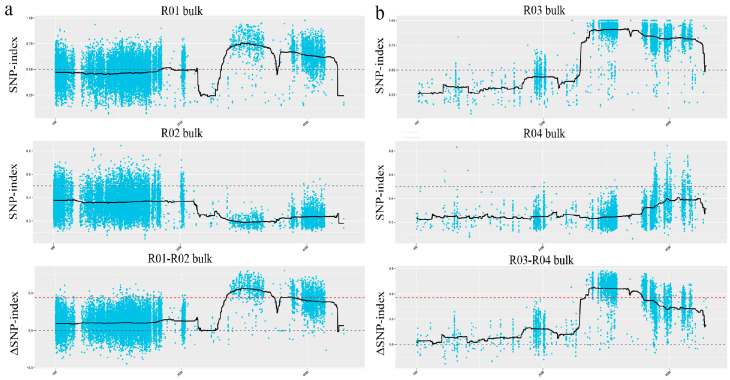
Distribution of SNP-index and ΔSNP-index on chromosome 13 in the highly resistant lines R47 and R55. The dashed line indicates the significance threshold for the SNP-index, while the solid black line shows the actual SNP-index values across chromosomal positions. (**a**) R47 highly resistant materials. (**b**) R55 highly resistant materials. The X-axis represents the physical position (Mb) on chromosome 13, and the Y-axis indicates the SNP-index and ΔSNP-index values, respectively.

We further conducted a focused analysis of chromosome 13 (Figure 3), which confirmed the presence of an overlapping candidate region in both the R47 and R55 mutants. This observation strongly suggests that SMV resistance in these two lines is likely controlled by mutations in the same gene.

### 2.3. Screening of Resistance Candidate Genes

**Figure 4:**
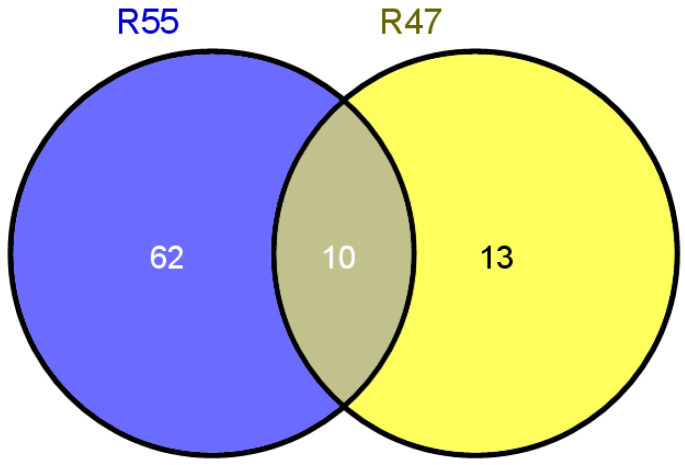
Venn diagram analysis of overlapping candidate resistance genes between the highly resistant lines R47 and R55.

Genes with SNP variations within the candidate resistance regions of the R47 and R55 mutants were systematically screened. Candidate genes were selected based on three primary criteria: (1) high expression levels in leaf tissues; (2) SNPs located in exons, splice sites, or promoter regions (which may affect gene expression); and (3) the results of prior research indicating involvement in biotic stress resistance. This screening identified 72 candidate genes in R55 and 23 in R47, with 10 genes shared between the two mutants (Figure 4). These 10 shared genes were subjected to functional annotation and expression profiling [38]. Among these, *Glyma.13G194900* was annotated as a resistance (*R*) gene and prioritized for further transgenic validation (Table 1). Sequence analysis showed that this gene has G-to-A transitions at nucleotide positions 122 and 166, leading to amino acid substitutions: glycine to glutamic acid (GGA→GAA) and valine to aspartic acid (GTT→GAT), respectively.

### 2.4. Transgenic Resistance Assay of the Candidate Resistance Gene

**Figure 5:**
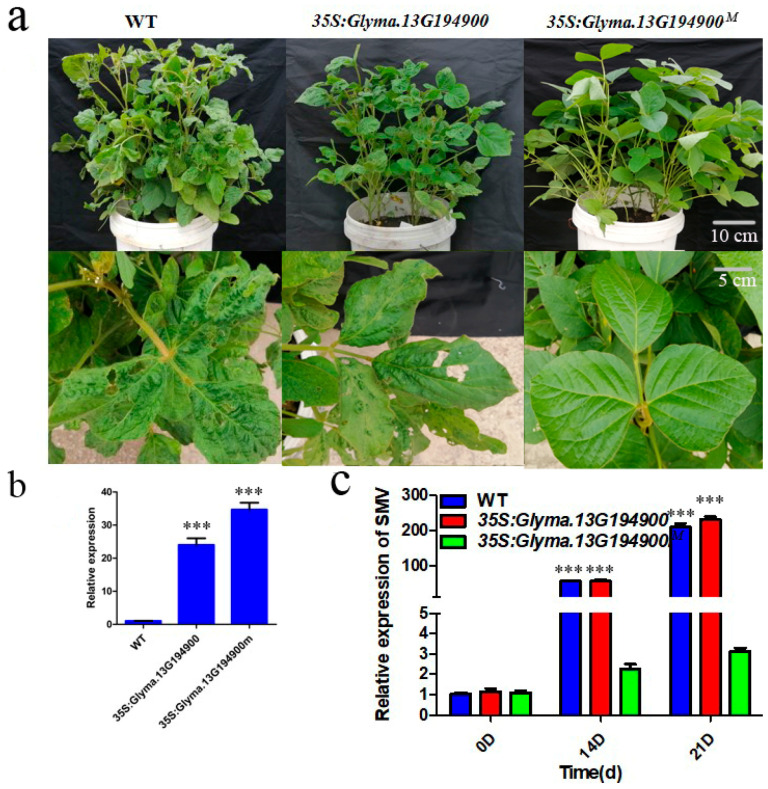
Overexpression of *Glyma.13G194900^M^* in soybean increases resistance to SMV. (**a**) Phenotypes of WT, *35S*:*Glyma.13G194900^M^*, and *35S*:*Glyma.13G194900* transgenic plants after SMV inoculation. The top three panels show the overall phenotypes of pot-grown plants, while the bottom three panels show close-up views of newly expanded unifoliate leaves. (**b**) Relative expression levels of *Glyma.13G194900^M^* and *Glyma.13G194900* in transgenic plants. Data represent the mean ± SD of three independent experiments, each with three technical replicates. (**c**) Virus accumulation in WT, *35S*:*Glyma.13G194900^M^*, and *35S*:*Glyma.13G194900* transgenic plants. Data represent the mean ± SD of three independent experiments at 14 and 21 days post-SMV inoculation, each with three technical replicates. The asterisks represent significantly different virus contents (Student’s *t*-test, ***, *p* < 0.001).

To validate whether *Glyma.13G194900* (WT gene) and *Glyma.13G194900^M^* (mutant gene) function in SMV resistance, we obtained transgenic overexpression materials for both genes. The T_1_ generation was self-pollinated to produce T_2_ transgenic lines, which included those overexpressing *Glyma.13G194900* and *Glyma.13G194900^M^*. Initially, the transgenic lines were screened using Bar test strips and herbicides. Quantitative real-time PCR (qRT-PCR) analysis of the overexpression lines showed that both genes were expressed at levels more than 20-fold higher than in the wild type (WT) before SMV inoculation (Figure 5b). At approximately 21 days after seedling emergence, transgenic plants and WT plants were inoculated with SMV-SC3. Tissue samples were collected at 14 and 21 days post-inoculation to detect SMV content.

A resistance assessment showed that plants overexpressing *Glyma.13G194900^M^* exhibited a highly resistant phenotype to SMV-SC3, whereas those overexpressing the *Glyma.13G194900* did not show enhanced resistance to SMV-SC3 (Figure 5a,c). This indicates that the mutation in *Glyma.13G194900* confers resistance to SMV-SC3, classifying it as a gain-of-function mutation.

### 2.5. Overexpression of Glyma.13G194900^M^ Inhibits Viral Replication and/or Cell-to-Cell Movement

**Figure 6:**
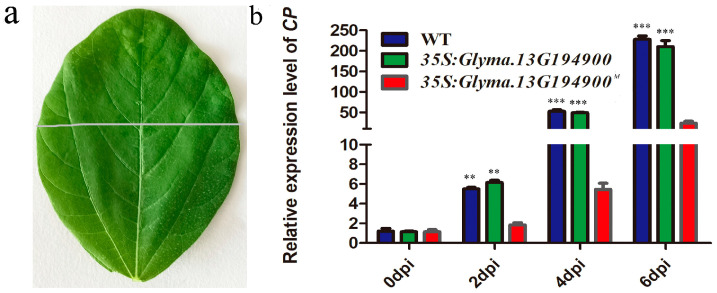
Overexpression of *Glyma.13G194900^M^* reduces SMV accumulation in soybean leaves. (**a**) Trifoliate leaves of 14-day-old WT, *35S*:*Glyma.13G194900^M^*, and *35S*:*Glyma.13G194900* transgenic plants were inoculated with SMV-SC3. The upper leaf section (above the white line) was inoculated with SMV, while the lower section (below the white line) of the same leaf were collected at 0, 2, 4, and 6 dpi. Total RNA was isolated for qRT-PCR analysis of viral RNA levels. (**b**) SMV content in WT, *35S*:*Glyma.13G194900^M^*, and *35S*:*Glyma.13G194900* transgenic plants at 0, 2, 4, and 6 dpi. Data represent the mean ± SD of three independent experiments, each with three technical replicates. The asterisks represent significantly different virus contents (Student’s *t*-test, **, *p* < 0.01, ***, *p* < 0.001).

To further investigate the mechanism by which *Glyma.13G194900^M^* overexpression confers SMV resistance, we inoculated the mature upper leaves of both *Glyma.13G194900^M^*-overexpressing (OE) lines and wild-type (WT) plants with the SMV-SC3 strain. Lower non-inoculated leaf samples were collected at 2, 4, and 6 days post-inoculation (dpi) to monitor viral spread (Figure 6a). Quantitative real-time PCR (qRT-PCR) detection of the viral coat protein (CP) gene revealed a statistically significant reduction in SMV accumulation in OE plants compared to WT controls at all time points (Figure 6b). This result indicates that overexpression of *Glyma.13G194900^M^* likely retards viral replication efficiency and/or blocks the cell-to-cell movement of SMV, thereby limiting systemic infection.

## 3. Discussion

As a critical global food, stable soybean production is crucial for ensuring food security and agricultural trade. However, the persistent threat of SMV remains one of the major biotic stress factors limiting high and stable soybean yields [3]. While traditional breeding has achieved notable success in identifying and utilizing disease-resistant genes, the process is time-consuming and often constrained by germplasm resource availability. However, with advances in functional genomics, techniques such as map-based cloning have accelerated the cloning of plant disease resistance genes [39]. Among these, MutMap has been widely applied to clone genes controlling key agronomic traits in crops.

Traditional map-based cloning requires the construction of large segregating populations and it relies on linkage analysis to gradually narrow down the target gene region, making it cumbersome and time-intensive. In contrast, MutMap leverages the high throughput of whole-genome resequencing to rapidly and accurately locate genes associated with target traits [35,36]. In this study, we used an M_3_ mutant population generated by EMS mutagenesis of Williams 82 [40], combined with field inoculation experiments using the SMV-SC3 strain (Figure 1 and Figure S1). EMS mutagenesis is known to induce abundant genetic variation, providing greater opportunities for gene screening [41]. Through whole-genome resequencing and bioinformatics analysis, we efficiently narrowed the candidate region to a genomic segment containing *Glyma.13G194900* (Figure 2). This process effectively circumvented the interference of homologous sequences caused by the high degree of genome duplication in soybean, highlighting the unique advantages of MutMap in studying crops with complex genomes [36].

A sequence analysis of the candidate gene *Glyma.13G194900* revealed two key missense mutations: G122E (glycine to glutamic acid) and V166D (valine to aspartic acid). Both of these mutations result in significant changes in amino acid properties—from non-polar (glycine, valine) to polar, charged residues (glutamic acid, aspartic acid)—which are highly likely to induce conformational changes in the protein’s three-dimensional structure and thereby alter its function [42]. The most critical evidence was derived from transgenic functional validation: introducing the mutant allele (*Glyma.13G194900^M^*) into susceptible soybean significantly enhanced resistance to SMV-SC3, whereas introducing the WT allele did not (Figure 5). This outcome unequivocally confirms that *Glyma.13G194900^M^* is a gain-of-function mutation. Furthermore, we observed that overexpression of *Glyma.13G194900^M^* likely slows down viral replication efficiency and/or blocks the cell-to-cell movement of SMV, thereby restricting systemic infection (Figure 6). We speculate that this increased binding affinity between this protein and viral effectors enhances effector-triggered immunity (ETI), resulting in increased programmed cell death [43].

Nevertheless, there are several scientific issues regarding *Glyma.13G194900^M^* that still need to be addressed. The first relates to the original biological function of the *Glyma.13G194900* gene and how the mutation rewires its molecular network to confer resistance need clarification. In this respect, techniques such as yeast two-hybrid assays to screen for interacting proteins will help elucidate the signaling pathways involved. Secondly, it is essential to evaluate evaluating the resistance spectrum of *Glyma.13G194900^M^* and systematic testing of its resistance to other prevalent SMV physiological strains is required. If the resistance spectrum is narrow, this gene should be combined with other identified broad-spectrum resistance genes via gene pyramiding strategies to develop elite varieties with durable, broad-spectrum resistance [44].

In conclusion, our results demonstrate that MutMap can rapidly identify genes associated with SMV resistance, providing valuable genetic resources to accelerate the development of SMV-resistant soybean cultivars.

## 4. Materials and Methods

### 4.1. Plant Materials and Virus

The soybean cultivar Williams 82 was used as both the transgenic recipient and the parent for ethyl methanesulfonate (EMS) mutagenesis. EMS-mutagenized materials were kindly provided by Researcher Xianzhong Feng from the Northeast Institute of Geography and Agroecology, Chinese Academy of Sciences [40]. From this mutant population, two lines (R55 and R47) with high resistance to SMV-SC3 were identified. All plant materials were grown in experimental fields in Gongzhuling, Jilin Province. Additionally, the binary expression vector *pCAMBIA3300-35S* and *Agrobacterium tumefaciens* strain EHA105 were maintained in our research group.

### 4.2. Gene Clone and Vector Construction

The candidate genes *Glyma.13G194900* (WT) and *Glyma.13G194900^M^* (mutant) were cloned using Williams 82 and R55/R47 mutant tissues as templates, respectively. The cloned genes were digested with the restriction enzymes Xba I and Sac I, then ligated into the binary expression vector *pCAMBIA3300-35S* (driven by the CaMV 35S promoter). The recombinant plasmid was introduced into *Agrobacterium tumefaciens* strain EHA105 via the freeze–thaw method.

### 4.3. Soybean Transformation and Screening of Transgenic Plants

Williams 82 was used as the transgenic recipient, and genetic transformation was performed using the *Agrobacterium*-mediated cotyledonary node transformation method [45]. Transgenic plants were initially screened using PAT/BAR test strips (following the manufacturer’s instructions). Subsequently, the target gene was verified in transgenic seedlings via PCR using gene-specific primers: *Glyma.13G194900*-F (5′-TCGGTCCGAGAAAACATATC-3′) and *Glyma.13G194900*-R (5′-TTTGTGAGCCTTTCTACCTC-3′).

### 4.4. Genetic Mapping and Bulked Segregant Analysis (BSA)

F_2_ populations derived from crosses between SMV-resistant lines (R47/R55) and Williams 82 were used for genetic mapping. Equal amounts of DNA from resistant and susceptible F_2_ individuals were pooled to create two DNA bulks: a “resistant bulk” and a “susceptible bulk.” Paired-end sequencing libraries were constructed for both bulks and sequenced on an Illumina HiSeq™ 2500 platform (Illumina, San Diego, CA, USA) at BIOMARKER Biotech Company (Beijing, China).

Single-nucleotide polymorphisms (SNPs) between the mutant bulks and Williams 82 were identified by aligning sequencing reads to the Glycine max Wm82.a4.v1 reference genome (https://phytozome.jgi.doe.gov/pz/portal.html, accessed on 15 December 2020). SNP filtering was performed using BWA (v0.7.17) (Burrows-Wheeler Aligner) and GATK (v4.1.9.0) (Genome Analysis Toolkit) software, Briefly, the BWA-aligned results were first processed with Picard’s MarkDuplicates (v2.23.7) to remove PCR duplicates. Then, base quality score recalibration (BQSR) was conducted using GATK to correct base quality scores. Finally, variant calling with GATK was carried out to identify SNPs and InDels. SNPs were filtered with the following criteria: (1) exclude heterozygous SNP loci in the parental lines; (2) remove SNP loci with consistent genotypes between parental and bulk samples; (3) filter out SNPs not induced by EMS (EMS typically induces G:C→A:T transitions); (4) exclude SNPs with a sequencing depth < 5× in the F_2_ bulks. Ultimately, 200,200 high-quality, reliable SNPs were retained. The ΔSNP-index was calculated as: ΔSNP-index = (SNP-index of the mutant bulk) − (SNP-index of the WT bulk). Genomic regions with a ΔSNP-index > 0.5 were designated as candidate regions [40].

### 4.5. Quantitative RT-PCR (qRT-PCR)

Total RNA was extracted from leaves of transgenic and WT plants using the EasyPure Plant RNA Kit (Transgen Biotech, Beijing, China). First-strand cDNA was synthesized using the ThermoScript RT-PCR System (Invitrogen, Carlsbad, CA, USA). qRT-PCR was performed with the following conditions: initial incubation at 50 °C for 2 min, followed by 10 min at 95 °C, and 35 cycles of 95 °C for 15 s, 60 °C for 30 s, and 72 °C for 30 s. GmActin11 was used as the internal reference gene (primers: *GmActin11*-F: 5′-ATCTTGACTGAGCGTGGTTATTCC-3′; *GmActin11*-R: 5′-GCTGGTCCTGGCTGTCTCC-3′). Each sample included three biological replicates, with three technical replicates per biological replicate.

### 4.6. Disease Resistance Assessment in Transgenic Plants

Transgenic lines and WT plants were inoculated with the SMV-SC3 strain at the first trifoliate leaf stage. One month post-inoculation, plant phenotypes were recorded, and viral content was determined via qRT-PCR using primers specific to the SMV *CP* gene: *CP*-F (5′-AACAGGGCAAGGGAAGCAAT-3′) and *CP*-R (5′-CCATGCCCAAAAGAGTGTGC-3′). Resistance was classified based on phenotypic symptoms (e.g., mosaic, necrosis) and relative viral CP gene expression levels.

For inoculation, SMV-SC3 was ground in liquid nitrogen and homogenized in 0.01 M phosphate buffer (pH 7.2–7.4) at a 1:10 (*w/v*) ratio. The inoculum was then applied to trifoliate leaves by gently abrading the surface with carborundum or a brush.

## Figures and Tables

**Table 1:** Functional annotation of candidate genes and their FPKM expression values in leaves.

Candidate Gene	Functional Annotation	New Leaf Tissue Expression Level (FPKM)
*Glyma.13G162200*	Translocase of chloroplast 90	1.7
*Glyma.13G178200*	RNA processing and modification	0.5
*Glyma.13G179000*	Signal transduction mechanisms	2.1
*Glyma.13G182200*	Glycosyl transferases group	0
*Glyma.13G182600*	RNA processing and modification	6.2
*Glyma.13G190000*	NB-LRR-ARC domain (R-gene)	0.3
*Glyma.13G191000*	LOB domain-containing protein	0
*Glyma.13G194800*	NB-LRR-ARC domain (R-gene)	1.3
*Glyma.13G194900*	NB-LRR-ARC domain (R-gene)	4.2
*Glyma.13G201800*	DNA Repair protein	0

## Data Availability

The original contributions presented in this study are included in the article/Supplementary Material. Further inquiries can be directed to the corresponding authors.

## Supplementary Materials

The following supporting information can be downloaded at: https://www.mdpi.com/article/10.3390/plants14223504/s1, Figure S1: Screening of ~14,000 M_3_ EMS-mutagenized soybean plants via three rounds of field inoculation with the SMV-SC3 strain.
