# Supplementary material for: MutMap-Based Cloning of a Soybean Mosaic Virus Resistance Gene
Source: Plants (Basel). 2025 Nov 17;14(22):3504. doi: 10.3390/plants14223504 (PMC12656595; doi:10.3390/plants14223504)
Supplement: Supplementary file 1 [file plants-14-03504-s001.zip › plants-3960481-supplementary.pdf]

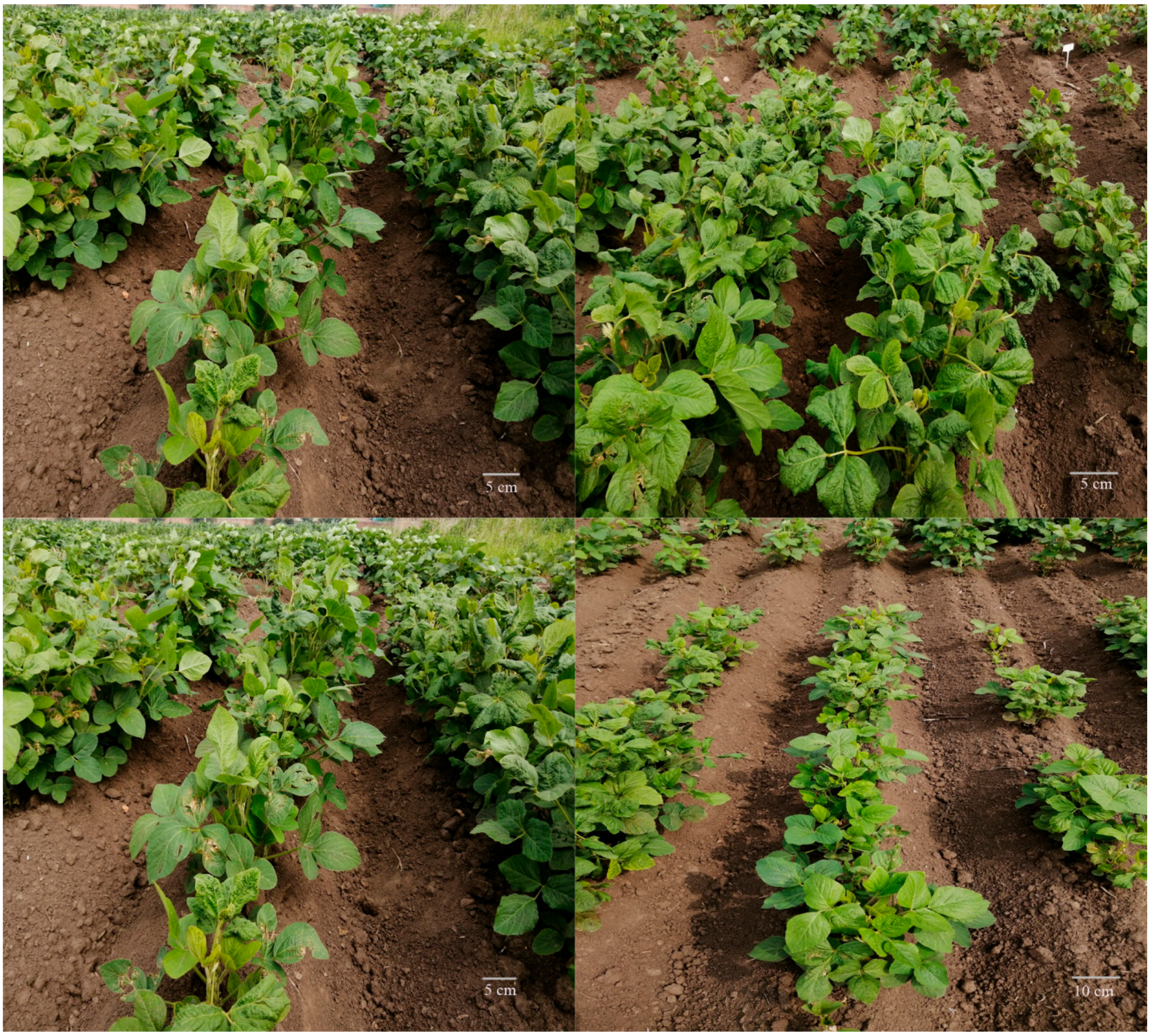

Figure S1. A screening of approximately 14,000 M_3 EMS mutant plants was conducted by performing three rounds of inoculation with SMV-SC3 strain in the field.
